# Comprehensive overview of management and risk assessment of breast cancer-related lymphedema: a multidisciplinary approach

**DOI:** 10.3389/fonc.2026.1796657

**Published:** 2026-05-19

**Authors:** Elisheva Knopf, Lily T. Childers, Mackenzie Woodward, Isabella M. Gray, Justin T. Childers, Sarfraz Ahmad

**Affiliations:** 1 Charles E. Schmidt College of Medicine, Florida Atlantic University, Boca Raton FL, United States; 2 College of Medicine, Florida State University, Tallahassee, FL, United States; 3 Orlando Health Jewett Orthopedic Institute, Orlando, FL, United States; 4 Gynecological Oncology Program, AdventHealth Cancer Institute, Orlando, FL, United States

**Keywords:** axillary lymph node dissection, breast cancer-related lymphedema, diagnosis, lumpectomy, lymphedema, malignancy, mastectomy, prevention

## Abstract

Breast cancer is one of the most commonly diagnosed cancers worldwide, with a particularly high incidence in women. Although many are cured of their malignancy, treatment options can present with significant long-term complications for the patient. Lymphedema is the accumulation of protein-rich fluid that occurs following a disturbance to the lymphatic system. Secondary lymphedema to the arm often results following breast surgery or axillary lymph node dissection, although risk and exacerbating factors include radiation and medical chemotherapy, both in the short- and long-term intervals. Breast cancer-related lymphedema (BCRL) develops secondary to treatment-related lymphatic injury, commonly involving the axillary lymphatic system, with clinical manifestations that may affect the ipsilateral arm, hand, breast, chest wall, and axilla. This article serves as an updated and comprehensive review of the available peer-reviewed literature regarding the pathophysiology, incidence, predictive/risk factors, clinical features, diagnostic strategies, comparative accuracy and utility of early detection and surveillance approaches, preventive strategies, and guidelines-based conservative/surgical/emerging management approaches for BCRL. The purpose of this review is to apprise healthcare professionals with the latest understanding of optimal care and counseling patients regarding BCRL.

## Introduction

In the United States, breast cancer is the most diagnosed cancer among women, the second-leading cause of cancer-related death in women, and represents a major public health burden (1). An estimated one in eight women (13.1%) in the United States will be diagnosed with invasive breast cancer during their lifetime (2). Female gender represents the strongest risk factor for breast cancer, with approximately 99% of cases occurring in women and only 0.5-1.0% occurring in men (3). Additional risk factors for the development of breast cancer include obesity, increasing age, alcohol and tobacco use, and family history of breast cancer (3). However, nearly half of breast cancers occur in women with no identifiable risk factors other than female gender and age (3). Although breast cancer risk increases with advancing age, recent trends demonstrate a growing incidence in invasive breast cancer, with the most pronounced increases occurring amongst younger women (2).

As a result, an increasing number of patients are exposed to current breast cancer treatments, which include surgery, radiation therapy, hormone therapy, and targeted systemic therapies (4). Long-term complications can arise following surgical, radiation, and systemic treatment, particularly when multiple approaches are combined (5). BCRL represents a major treatment-related complication of breast cancer, with risk strongly associated with axillary lymph node dissection (ALND) and regional lymph node radiation (RLNR) (6). BCRL is a chronic condition resulting from lymphatic system damage that leads to impaired lymphatic drainage in the affected breast region and into the arm, hand, and/or breast (7). Approximately one in five (~20%) patients treated for breast cancer develop BCRL, which is associated with symptoms including arm or hand swelling, pain, and heaviness (6, 7). Additionally, BCRL can lead to more serious infectious complications, including soft tissue infections such as cellulitis (7, 8). Beyond its clinical complications, BCRL contributes to psychological morbidity and reduced health-related quality of life (HRQoL), and is associated with increased out-of-pocket healthcare costs (9, 10). Despite advances in breast cancer care, BCRL remains under-recognized and undertreated in clinical practice (11).

This manuscript is a narrative review designed to provide a multidisciplinary, clinically focused update on BCRL. A targeted literature search was conducted using PubMed, Scopus, and major oncology and surgical oncology journals to identify peer-reviewed studies addressing pathophysiology, incidence, risk stratification, diagnostic modalities, surveillance strategies, prevention, and management approaches. Emphasis was placed on recent literature, landmark trials, guideline statements, and studies with direct clinical applicability. Articles were selected based on relevance to current clinical practice, methodological rigor, and clinical applicability to provide a comprehensive and practice-oriented synthesis of data. This review was not conducted as a systematic review and therefore does not follow PRISMA guidelines. The goal of this review is to provide clinicians with an updated framework reflecting current evidence and evolving strategies in BCRL care. A 2024 review provided a comprehensive overview of the diagnosis, prevention, and treatment of post-mastectomy lymphedema (12). Since that publication, the literature has expanded to include updated risk stratification models, refined staging classifications, evolving surveillance frameworks, comparative evaluations of early detection modalities, expanded cost-effectiveness analyses, and updated guideline recommendations. The present review incorporates these developments and extends prior work by providing a multidisciplinary synthesis that emphasizes risk assessment, longitudinal surveillance, and contemporary preventive and management strategies.

## Pathophysiology

**Figure 1:**
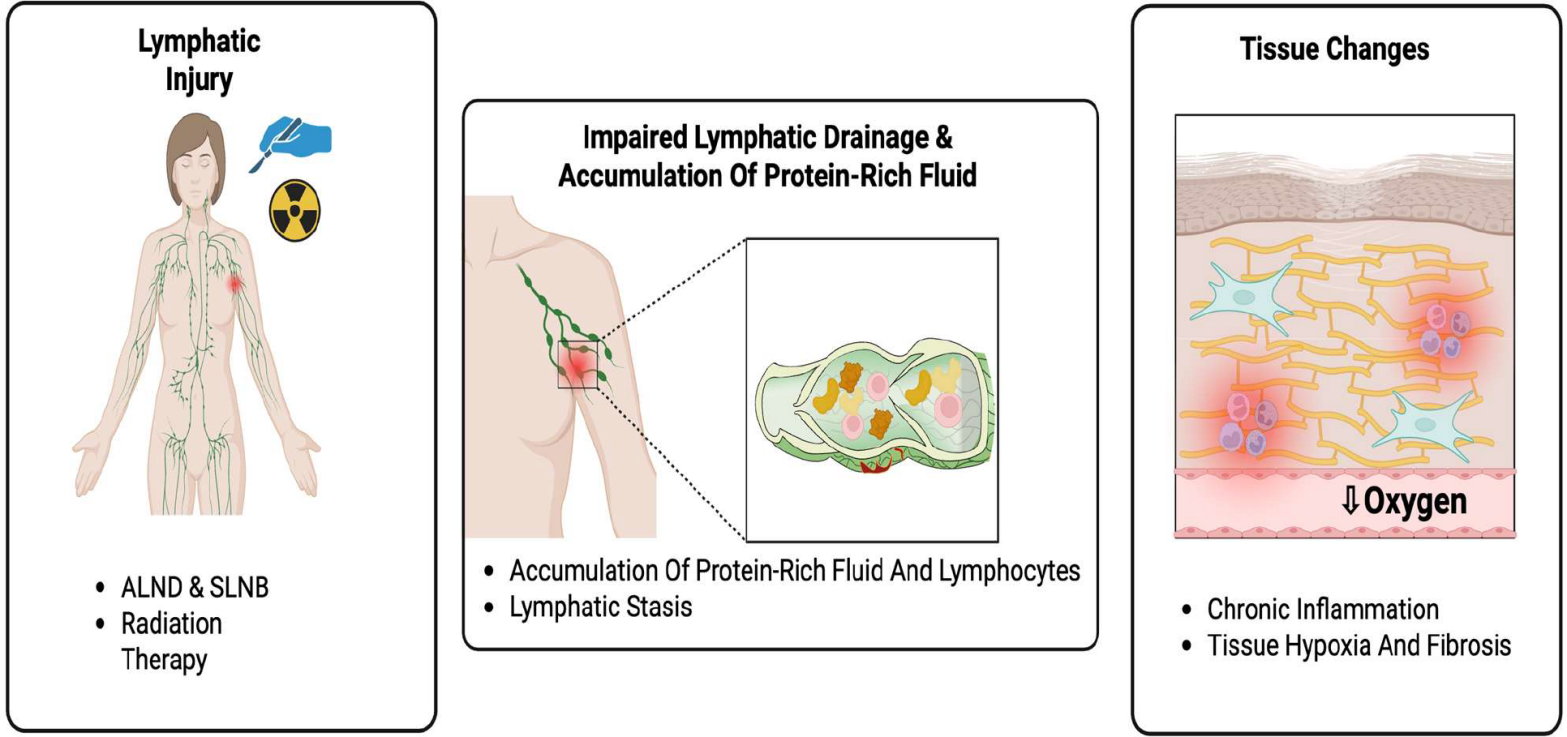
Schematic representation of the pathophysiologic mechanisms underlying breast cancer-related lymphedema (BCRL). ALND, Axillary Lymph Node Dissection; SLNB, Sentinel Lymph Node Biopsy; BCRL, Breast Cancer-Related Lymphedema.

BCRL results from impaired lymphatic transport following damage to the lymphatic system, leading to accumulation of protein-rich fluid in the interstitial tissue (12, 13). This protein-rich fluid accumulation triggers a cascade of inflammation, tissue fibrosis, and adipose tissue expansion (14). The lymphatic stasis creates a microenvironment characterized by chronic inflammation, progressive fibrosis, and tissue hypoxia, which further reinforces lymphatic failure and perpetuates the condition. The lymphatic system normally drains fluid and proteins from tissues back to the venous circulation. Surgical disruption through ALND or sentinel lymph node biopsy (SLNB), combined with radiation-induced vascular damage, impairs this drainage capacity (13, 15). **Figure 1** illustrates the pathophysiologic cascade underlying BCRL, beginning with lymphatic injury from surgical and radiation-based interventions and progressing to impaired lymphatic drainage, protein-rich fluid accumulation, and subsequent chronic inflammation, tissue hypoxia, and fibrosis (**Figure 1**).

The progression of lymphedema follows distinct stages that reflect increasing severity and decreasing reversibility (16, 17). As summarized below, stages 0 and 1 are reversible with early intervention, whereas stages 2 and 3 cannot be reversed, although with early intervention, disease progression can be prevented (17).

- Stage 0 (latent/subclinical): Lymphatic dysfunction without visible swelling. Subtle symptoms such as heaviness or fatigue may be present.
- Stage 1: Accumulation of fluid and protein causing pitting edema. Additional symptoms include increased girth, heaviness, and stiffness. In this stage, swelling is relieved with limb elevation.
- Stage 2: Spongy tissue consistency with less evident pitting as fibrosis develops. The tissue hardens and increases in size. In this stage, swelling is not relieved with elevation. Stage 2 can further be sub-classified as Stages 2a and 2b (18):
  • Stage 2a: Edema becomes non-pitting and irreversible with limb elevation; fibrotic tissue changes begin in this stage.
  • Stage 2b: Fibrosis and skin changes become more evident in this stage.
- Stage 3 (lymphostatic elephantiasis): Severe dry, scaly, thickened skin is present. There is marked swelling and girth. Fluid leakage and blisters are common as well. Pitting may be absent due to progressive fat deposition and fibrosis.

## Established risk factors

### Treatment-related factors

ALND represents the strongest treatment-related risk factor for BCRL, with 5-year cumulative incidence rates of 24.9% for ALND alone and 30.1% when combined with RLNR (19). Other studies similarly estimate the risk following ALND to range between 20-30% (20, 21). In contrast, SLNB carries substantially lower risk (8% for SLNB alone, 10.7% for SLNB with RLNR), though the risk still remains present (19).

Regional nodal irradiation independently increases lymphedema risk, particularly when targeting the breast, axilla, and supraclavicular regions (22, 23). The number of positive lymph nodes, especially more than eight, correlates with increased risk (15). Taxane-based chemotherapy has also been identified as an independent risk factor in multiple studies (15, 23).

Mastectomy vs. lumpectomy also significantly impacts lymphedema risk, with mastectomy being associated with a 42% increased risk compared to lumpectomy (RR 1.42, 95% CI 1.15-1.76) (24). A nationwide Korean study found 12.2% overall lymphedema incidence, with rates varying by surgical approach: 8.0% for breast-conserving surgery (BCS) with SLNB, 10.7% for total mastectomy with SLNB, 23.5% for BCS with ALND, and 28.5% for modified radical mastectomy (25).

Breast reconstruction has also been associated with a reduced risk of lymphedema. A 2025 meta-analysis of 15,670 patients found that those undergoing breast reconstruction had a significantly lower risk of lymphedema compared to those without reconstruction (incidence rate ratio 0.58, 95% CI 0.38-0.87, p<0.001) (26). Additional studies have reported similar findings, with immediate implant-based reconstruction following mastectomy associated with decreased lymphedema risk compared to mastectomy without reconstruction (27).

### Patient-related factors

Body mass index (BMI) ≥ 30 kg/m² significantly increases BCRL risk (23, 28). Obesity not only predisposes to lymphedema development but also complicates management, making weight loss an important preventive recommendation for overweight or obese patients (29).

A sedentary lifestyle has also been associated with increased risk (22). Additional established risk factors include localized infection, seroma formation, and postoperative complications related to surgical wounds or drains (15). Ipsilateral venous compromise from indwelling ports, pacemakers, or thrombosis may also contribute to BCRL (15).

## Emerging risk predictors

### Genetic factors and molecular biomarkers

Recent advances have broadened the understanding of the genetic basis of lymphedema, particularly in primary forms, though genetic factors and molecular biomarkers are emerging areas of investigation for secondary lymphedema risk prediction (14). Patient-specific biological factors are increasingly recognized as necessary components for comprehensive risk assessment beyond traditional treatment-related variables (30).

### Body composition metrics

Mammographic breast density has been identified as a novel predictive factor in recent risk modeling (30). Body composition metrics beyond simple BMI measurements are being explored to better characterize individual risk profiles (28).

### Surgical technique variations

Variations in surgical technique, including operative time, extent of axillary dissection (level I, II, or III), and surgical site infection rates, have been incorporated into newer predictive models (31). The number of lymph nodes removed correlates with the disease recurrence risk, independent of whether formal ALND was performed (31).

### Cumulative risk model

Risk prediction for BCRL requires individualized assessment incorporating multiple treatment-related, patient-specific, and biological factors (30). Traditional models based solely on cancer and treatment characteristics have shown limited accuracy when externally validated, with area under the curve (AUC) values often declining from approximately 0.7 in development cohorts to 0.6 in external validation (30).

More comprehensive models combining cancer treatment data with patient characteristics demonstrate improved predictive accuracy. A validated 5-factor model incorporating age, BMI, breast density, nodal burden, and use of ALND achieved sensitivity of 0.83, specificity of 0.89, and overall accuracy of 0.88 for predicting 2-year lymphedema-free survival (30). Other validated nomograms incorporating BMI, operative time, lymph node count, axillary dissection level, surgical site infection, and radiotherapy have shown AUCs of 0.779 in training cohorts and 0.724 in validation cohorts (31).

The multifactorial nature of BCRL risk necessitates personalized risk stratification to identify high-risk individuals who would benefit from closer surveillance, early intervention protocols, and preventive management strategies (22, 30). Current evidence supports that early detection through prospective surveillance and timely intervention can reduce progression to chronic lymphedema and limit tissue damage (32).

## Clinical features

BCRL presents with a spectrum of clinical manifestations that evolve over time. Swelling on the same side as cancer treatment is the universal symptom of lymphedema (33). However, the clinical presentation extends beyond visible edema to encompass a range of subjective and objective findings. Early symptoms are often subtle and can precede objective swelling, including sensations of heaviness, fatigue, fullness, or tightness in the affected limb (28). These symptoms can occur even in the absence of measurable volume changes, consistent with subclinical (Stage 0) lymphedema (34). Patient-reported symptoms such as perceived arm swelling has been shown to precede objective lymphedema by several months, with increased arm size appearing a median of 6.1 months before the onset of objective disease (35). The symptoms of “heaviness in the past year” and “swelling now” have been shown to predict measurable lymphedema with a maximal limb circumference difference of 2 cm (36). As lymphedema progresses, tissue changes become more apparent, with increasing limb volume, decreased range of motion, skin thickening, functional limitations, and development of fibrosis (28). General disease progression generally occurs over months to years, though the rate may vary depending on individual risk factors and the extent of lymphatic injury (15).

### Impact on employment

BCRL significantly impacts work productivity and employment status. Among working women with BCRL, 52.2% reported that lymphedema affected their careers, with the impact most pronounced in those with severely impaired arm movement (41, 42). The condition creates substantial occupational challenges, with 35% of women considering the global impact on arm use during professional activities to be high (42). For women with high vs. low arm-use occupations, multivariate analyses demonstrated that intermediate arm-use level was associated with significantly higher global impairment, as was high arm-use level (42). Furthermore, women whose jobs required constant use of the affected arm suffered profoundly from having BCRL (43).

The most common work-related complaints involve difficulty with elevation of upper limbs, carrying heavy objects, driving, and holding manual movements (44). These physical limitations lead to difficulty or impossibility of performing work tasks, diminished work productivity, and increased time to return to work (44). Studies suggest higher unemployment rates and the need for modifying work conditions among breast cancer survivors with lymphedema (44). Furthermore, patient-reported BCRL is associated with higher rates of absenteeism and work/activity impairment (45). Despite the significant impact, workplace adaptations remain rare but beneficial. Only 26.9% of patients received workplace adaptations, most commonly ergonomic modifications, though these were predominantly provided to those with greater arm-movement impairment (42).

### Psychosocial burden

BCRL exerts a profound impact on HRQoL, affecting physical, psychological, and social domains (37–39). In a regional population-based study assessing HRQoL with the Short Form-36 Health Survey (SF-36), the Lymphedema Functioning, Disability and Health Questionnaire (LYMPH-ICF), and the Disabilities of the Arm, Shoulder and Hand questionnaire (DASH), patients with BCRL reported significantly worse outcomes than those without lymphedema across the majority of assessed HRQoL domains (38). Within the SF-36 domains specifically (0–100 scale, higher scores indicating better HRQoL), BCRL was associated with large mean differences in physical functioning (27 points), mental health (24 points), and social role functioning (20 points) (38). These impairments may persist long-term, with significant HRQoL differences reported up to 10 years after breast cancer treatment (38). Notably, BCRL diagnosis appeared to drive larger decrements in HRQoL than clinical severity measures alone, suggesting substantial variability in coping and perceived disability (38).

Symptom burden plays a central role in patient experience and may better reflect disease impact than limb size alone. In the HEAL cohort study, symptoms such as tension, heaviness, burning pain, and warmth/redness were associated with poorer HRQoL as demonstrated with the Wesley Clinic Lymphedema Scale, and lower SF-36 Physical Component Summary (PCS) scores (40). Increasing symptom burden was also associated with poorer perceived physical health and higher perceived stress (40).

The psychosocial burden of BCRL is substantial, with higher rates of depression, emotional distress, and poorer overall health among reported patients (46). In a prospective cohort study, women with lymphedema demonstrated a 14% higher risk for scoring one level higher on mood disturbance tests, a 9% higher probability of scoring one point lower on HRQoL scales, and a 29% higher likelihood of reporting poorer or bad health compared to women without lymphedema (47). Negative emotions experienced by women with lymphedema commonly include anxiety, frustration, sadness, anger, fear, and increased self-consciousness (39). The fear of developing lymphedema is pervasive, with approximately 73% of patients reporting fear of BCRL; however, positively associated with improved adherence to preventative behaviors (45).

Lymphedema-related distress is further associated with worse mental health outcomes. Patients reporting moderate to severe distress had a 50% and 73% higher odds of reporting poor physical health and having poor mental health compared to women without lymphedema, respectively (7). Furthermore, patients with lymphedema were also more disabled, as measured by the World Health Organization Disability Assessment Schedule II (WHO-DAS II), with an overall disability score of 39.78 compared to 34.67 (48). Qualitative studies reveal that women with BCRL experience a “drastic change that affects all areas of their lives,” requiring adapting their current practices and searching for resources to assist in overcoming the condition (49). Patients often adopt coping mechanisms to manage lymphedema, including modifying activities, covering the affected limb, or adjusting work responsibilities (50). Supervisors and colleagues play an important role in maintaining identity and normalcy amongst patients suffering from this condition (50).

## Diagnostic strategies

### Clinical examination

#### Subjective and objective symptoms

Early subjective symptoms of BCRL include sensations of heaviness, tightness, fullness, or fatigue in the affected limb, which may occur in the absence of measurable swelling (“subjective lymphedema”) (15, 51). Symptoms can involve the arm, hand, breast, chest wall, or axilla and may progress over time (15, 40).

Objective symptoms vary by disease stage, with early BCRL characterized by soft, pitting edema and later stages demonstrating fibrotic, non-pitting changes with associated skin thickening (15). Because early clinical may be subtle or absent, reliance on symptoms and physical examination alone may delay diagnosis (15).

#### Screening intervals

Screening intervals may be individualized based on patient risk, institutional resources, and surveillance modality; however, current guidelines emphasize prospective surveillance with baseline measurements to facilitate early detection (52). Because clinically apparent lymphedema may develop in the absence of patient-reported symptoms, surveillance is recommended for breast cancer survivors regardless of symptom presence (52). The 2023 International Society of Lymphology Consensus Document further supports ongoing clinical surveillance and early symptom recognition in at-risk patients, but does not define standardized screening intervals. Instead, there is emphasis on individualized monitoring strategies based on patient risk profile and clinical context (53).

### Diagnostic tools

Historically, BCRL was diagnosed based on visible increases in limb circumference, reflecting more advanced stages of disease. In contrast, contemporary approaches emphasize prospective surveillance using objective measurement tools to identify subclinical fluid changes before overt volume expansion occurs (12).

#### Tape measure

Circumferential tape measurement is one of the most commonly used methods for quantifying lymphedema, relying on standardized limb circumference measurements to assess changes in limb size over time. It is widely used because it is easy to implement; however, it is time-consuming and dependent on each individual examiner’s technique, which can lead to variability between measurements (54).

#### Perometry

Perometry provides automated limb volume assessment with greater reproducibility and less operator dependence than circumferential tape measurement. It allows precise longitudinal measurement and localization of swelling and is therefore frequently used as a reference standard in clinical research. However, its routine use in surveillance is limited by cost and availability (54).

#### Bioimpedance spectroscopy

Bioimpedance spectroscopy (BIS) provides a non-invasive, objective assessment of extracellular fluid changes and can detect subclinical lymphedema before overt volume increases occur. The technique is rapid, repeatable, and well-suited for longitudinal surveillance in at-risk patients. However, BIS is limited when baseline measurements are unavailable, as well as when patients have bilateral limb disease, since the measurements rely on comparison between limbs (54).

#### Tissue dielectric constant

Tissue dielectric constant (TDC) measurement provides a rapid, non-invasive assessment of localized tissue water content and can detect regional changes not captured by limb-based measurements (55). Although studies demonstrate good reliability and correlation with clinical measures of swelling, standardized diagnostic thresholds have not been established, and availability remains limited (56).

#### Water displacement method

The water displacement method, also known as water volumetry, is a direct volumetric measurement technique for BCRL. It involves immersing the affected limb in a calibrated container of water, followed by measuring the volume of displaced water (57). The volume of displaced water corresponds to the volume of the submerged segment. Although this method is relatively inexpensive and conceptually simple, it requires full limb immersion, which may not be practical for patients with limited mobility (58). Furthermore, accuracy depends on careful collection and measurement of displaced water, making this technique susceptible to human error, which limits its use in clinical practice (58).

#### Ultrasound

Ultrasound assesses tissue composition and structural changes associated with BCRL but has variable diagnostic accuracy and limited sensitivity for early disease states. Consequently, its role in routine surveillance remains adjunctive (56).

**Figure 2:**
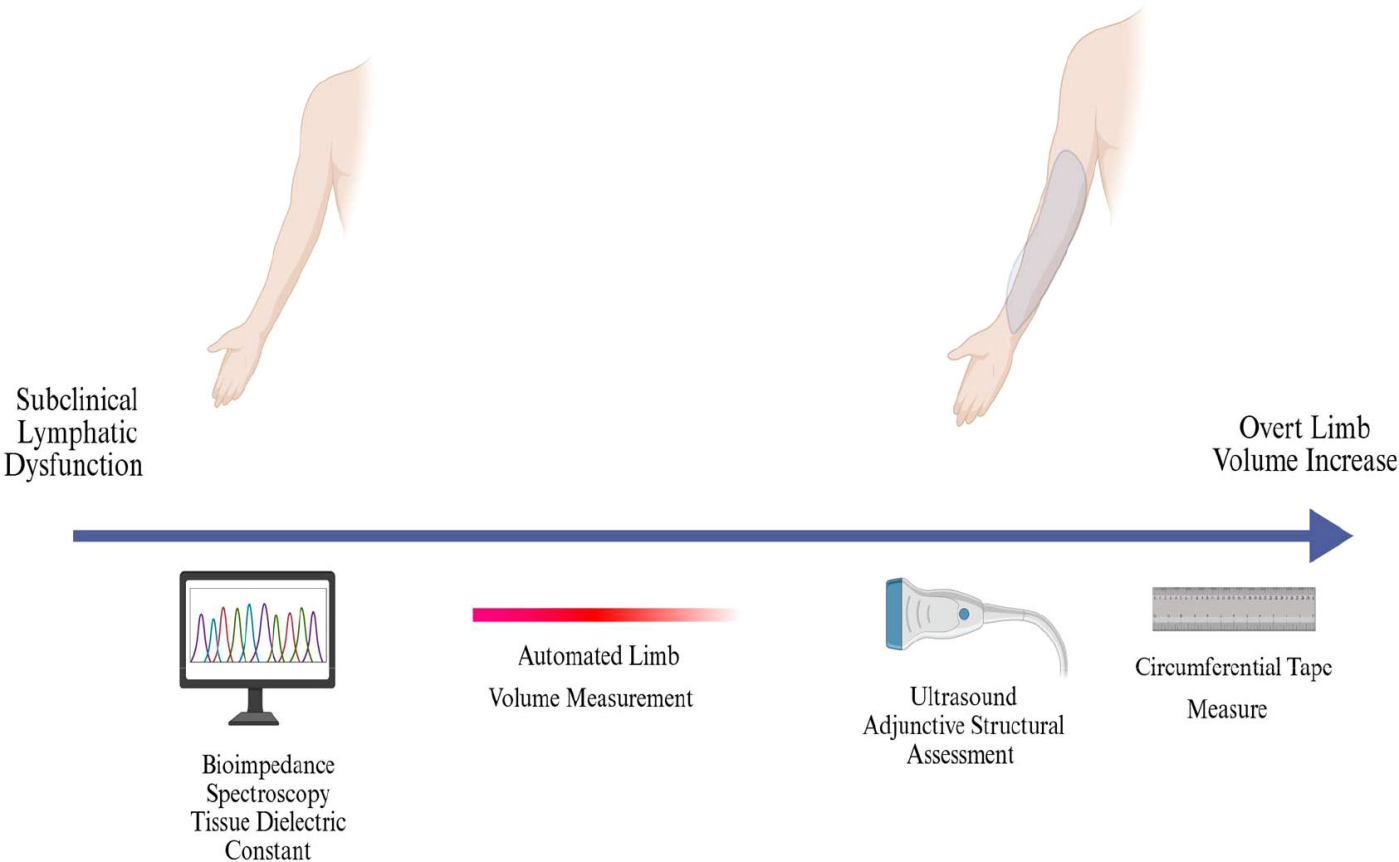
Diagnostic tools timeline for breast cancer-related lymphedema (BCRL).

**Figure 2** illustrates the temporal role of diagnostic modalities in BCRL, highlighting that techniques such as bioimpedance spectroscopy and tissue dielectric constant measurements can detect subclinical lymphatic dysfunction, whereas volumetric and circumferential measurements are more commonly utilized once overt limb volume changes are present (**Figure 2**). This underscores the importance of early surveillance strategies for identifying disease prior to clinically apparent swelling.

## Comparative accuracy and utility

Comparative studies evaluating surveillance strategies for BCRL demonstrate meaningful differences in the timing of detection and subsequent clinical outcomes across measurement modalities. The PREVENT randomized controlled trial showed that surveillance using BIS was associated with lower rates of progression to chronic lymphedema compared with monitoring based on circumferential tape measurement (59). Early identification of subclinical disease allowed for timely intervention, which reduced progression to clinically apparent lymphedema.

Importantly, the PREVENT trial also demonstrated that the risk of lymphedema extends beyond the immediate postoperative period, with new cases continuing to emerge several years after treatment. These findings highlight the importance of longitudinal surveillance rather than time-limited monitoring and support the role of early detection strategies in reducing disease progression over time (59).

## Cost-effectiveness of early detection

BCRL is associated with a substantial long-term financial burden, contributing to increased healthcare utilization and out-of-pocket costs for survivors. Affecting up to 35% of individuals treated for breast cancer, lymphedema is a chronic condition that can impair daily functioning and limit employment. Prior studies have shown that the cost associated with lymphedema are substantial, where patients with BCRL incur up to 112% of higher out-of-pocket costs compared to those without lymphedema (60). These sustained economic impacts highlight the importance of strategies aimed at preventing disease progression and reducing long-term financial burden/toxicity.

In this context, early detection through prevention-oriented surveillance has gained increasing attention. Recent evidence supports the use of BIS to identify subclinical disease and enable earlier, lower-intensity intervention before progression to chronic lymphedema (59). Cost-effectiveness analyses further suggest that BIS-based surveillance is associated with lower per-patient costs than tape measurement, largely due to reduced progression to more resource-intensive stages of disease (59).

## The NCCN and MASCC guidelines

### Screening intervals

**Figure 3:**
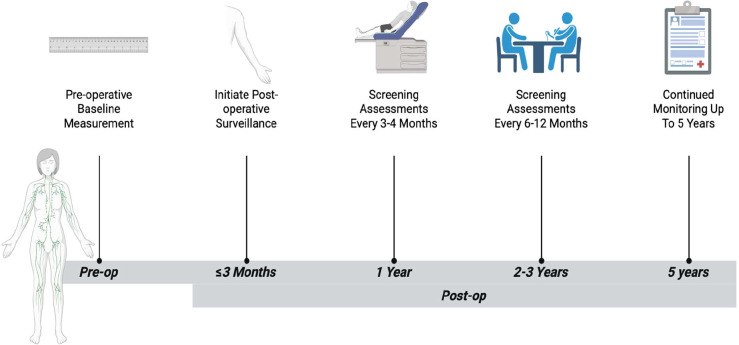
Surveillance timeline for breast cancer-related lymphedema (BCRL).

The National Comprehensive Cancer Network (NCCN) recommends routinely assessing patients for lymphedema-related symptoms and obtaining objective measurements when available to facilitate early detection (61). More detailed surveillance guidance is provided by the Multinational Association of Supportive Care in Cancer (MASCC), which recommends obtaining preoperative baseline measurements, initiating surveillance within three months following surgery, and continuing assessments every 3–4 months during the first postoperative year (62). Surveillance may then be extended to every 6–12 months for at least 2–3 years, with some guidelines supporting continued monitoring for up to five years after surgery (34, 62). **Figure 3** illustrates the recommended surveillance timeline for BCRL, emphasizing the importance of establishing preoperative baseline measurements, initiating early postoperative monitoring, and maintaining regular follow-up assessments over time (**Figure 3**). This longitudinal approach supports early detection of subclinical disease and enables timely intervention to reduce progression to clinically apparent lymphedema.

### Bioimpedance spectroscopy as an early detection option

The NCCN recommends BIS as an early detection option for BCRL in its 2025 Survivorship guidelines, noting that objective measurements, including BIS, may be obtained when available to identify early disease (61). In contrast, MASCC recommends BIS as an optional screening modality rather than a standard of care following publication of the PREVENT trial (62). This position reflects ongoing concerns regarding comparative performance relative to lower-cost methods and the potential for overestimation of lymphedema incidence. Although the PREVENT trial demonstrated lower progression rates with BIS-based surveillance (7.9% vs 19.2%), the MASCC noted possible imbalances in baseline risk factors between the study arms that may have influenced outcomes (62).

## Prevention strategies

### Lifestyle and behavioral interventions

Lifestyle and behavioral interventions represent foundational strategies in the prevention and risk minimization of BCRL, particularly among patients at elevated risk following axillary surgery. A recent systematic review evaluating aerobic and resistance exercise interventions supports the inclusion of structured exercise as part of a lifestyle modification prevention strategy, demonstrating potential benefits in mitigating early lymphatic destruction (63). In parallel, a large cohort study reported that supervised resistance training was associated with improved extracellular fluid balance amongst breast cancer survivors (64). These findings support the incorporation of structured, progressive exercise programs into lymphedema risk reduction strategies.

Elevated BMI is consistently associated with increased risk of BCRL, with recent analysis reaffirming obesity as a significant risk factor (65). This emphasizes the importance of early identification of at-risk patients and incorporation of weight management strategies into survivorship care to mitigate BCRL risk and progression.

Cellulitis, a common soft tissue infection in patients with impaired lymphatic function, represents a clinically important complication that contributes to lymphedema progression, recurrence, and increased morbidity in BCRL, emphasizing the importance of infection avoidance as a preventative strategy (66).

### Early postoperative physical therapy

Initiation of early (less than four weeks) postoperative physical therapy has been associated with improved shoulder function compared to routine care, suggesting that early structured rehabilitation may facilitate patient recovery and minimize risk factors associated with BCRL. Emerging evidence highlights that despite data supporting the benefits of early rehabilitation on preventing persistent stiffness and functional limitations, delayed implementation and hesitance leading to underutilization are common (67).

Recent evidence supports the integration of gradual, structured resistance training among breast cancer survivors, highlighting that progressive strength exercise may improve lean mass and fluid balance in the upper extremities. More recently, a cohort study demonstrated physiologically favorable changes in extracellular fluid measures, supporting the incorporation of resistance training as a mitigation strategy (50). These findings align with contemporary understandings of exercise oncology, which advocate for individualized, gradually escalated resistance training regimens in patients at risk for BCRL (64).

### Manual lymphatic drainage

Manual lymphatic drainage (MLD) is a specialized, gentle, skin-stretching technique intended to augment superficial lymphatic uptake and redirect lymphatic territory (68). It is most utilized as a component of complex decongestive therapy (CDT) in BCRL. CDT is currently recognized as the gold-standard, multi-component treatment for lymphedema, often combining such techniques as gentle massage, compression, skin care, and targeted exercises to minimize lymphedema (68). Although MLD has been evaluated as part of multimodal conservative interventions for BCRL, there is no definitive evidence published within the last year or so demonstrating that MLD alone reduces the incidence of lymphedema when used prophylactically in at-risk postoperative patients. Recent evidence supports the feasibility and clinical utility of MLD when embedded within standardized protocols.

A recent non-inferiority randomized controlled trial in patients with BCRL included MLD into a self-administered CDT program (69). The program regimen also consisted of compression, exercise, skin care, and education and was found to be not inferior to therapist-administered CDT for limb outcomes and health-related measures at short-term follow-up (69). During the three-month follow-up, the therapist administered group had a non-significant increase in severity, while the self-administered group continued to show improvement. An earlier review reports that CDT is associated with improvements in limb volume and symptoms, although the incremental contribution of manual lymphatic drainage remains inconsistent across the studies due to methodological heterogeneity (69). Notably, symptom-based outcomes, including limb heaviness and discomfort, demonstrated more consistent improvement than objective volume measures (70).

### Compression-based prophylaxis

Compression-based prophylaxis has been evaluated as a preventative strategy for BCRL for years regarding high-risk patients. A randomized controlled trial demonstrated that prophylactic use of a compressive sleeve during the first postoperative year following axillary lymph node surgery significantly reduced the incidence of arm swelling and delayed time to onset compared with standard care, supporting the use of compression as a primary preventative intervention in these populations (71). In another randomized controlled trial (72), early application of light compression sleeves after axillary lymph node intervention was associated with reduced early postoperative arm swelling and lower rates of early lymphedema compared with the standard of care. The intervention group wore a light compression sleeve early after surgery compared with the control group, which received standard postoperative care. Outcomes measured included arm circumference, incidence of arm swelling, and early manifestation of lymphedema. Though no new research has been definitively able to isolate compression as a form of BCRL prophylaxis in the last year, these findings support the role of early compression in reducing risk of BCRL (72).

### Surgical intervention as prophylaxis

Surgical prophylaxis in more recent years has been integrated at the forefront of lymphedema prevention (73). Immediate lymphatic reconstruction (ILR), most performed using lymphaticovenous anastomosis at the time of ALND, often referred to as LYMPHA (lymphatic microsurgical preventive healing approach), has been developed as a primary surgical intervention to minimize BCRL. The technique was first recognized in prospective clinical studies to be a comparable method of reducing postoperative lymphedema incidence following axillary dissection alone (74). Prospective and randomized clinical studies support that ILR performed at the time of lymph node dissection is associated with decreased early incidence rates of BCRL compared to axillary dissection alone (75).

Other methods of prophylactic surgical intervention intended to minimize BCRL include preventative microsurgical procedures, such as the prophylactic lymphaticovenous bypass. This procedure is performed to reduce the risk of BCRL in high-risk patients undergoing axillary intervention (76). A systematic review evaluating this procedure in patients receiving the prophylactic surgery reported a lower incidence of lymphedema compared to historical controls (63). However, heterogeneity in surgical technique and patient selection limited direct comparison across the studies (77). Institutional series further supported that prophylactic bypass performed at the time of axillary node dissection is associated with reduced BCRL incidence (78).

Reported outcomes following these prophylactic lymphatic surgical procedures suggest that these interventions are well tolerated by patients, with low rates of procedure-isolated morbidity when performed during primary oncologic surgery (79). Early prospective studies on LYMPHA reported no significant increase in infections, wound complications, or morbidity directly attributable to the lymphedema prophylaxis procedures (80). Subsequent follow-up studies demonstrated that lymphatic bypass patency is maintainable for several years, though some patients did experience delayed symptom progression requiring another form of conservative therapy (81).

### Breast cancer reduction as prophylaxis

Optimization and prompt treatment of breast cancer, particularly axillary management, plays a key role in reducing the incidence of BCRL. SLNB is associated with a significantly lower incidence of postoperative BCRL when compared with axillary lymph node dissection. The SLNB patients present with lower rates of BCRL after extensive axillary surgery (82). Meta-analysis evidence supports that SLNB offers equal oncologic benefits compared to standard axillary dissection while also reducing postoperative morbidity and dysfunction (83). Recent data indicates that the risk of BCRL is influenced by the extent of lymph node removal and treatment method, such as differences in radiation therapy and systemic chemotherapy, supporting tailored axillary surgical strategies designed to minimize lymphatic invasion (84). All strategies to prevent BCRL are summarized in **Table 1**.

**Table 1:** Prophylactic strategies for prevention of breast cancer-related lymphedema (BCRL).

Intervention category	Timing relative to axillary surgery	Primary preventive intent	Key outcome
Lifestyle and Exercise	Pre- and post-operative	Risk Reduction	↑ fluid balance; ↓ risk (64–66)
Early Physical Therapy	Post-operative	Functional Recovery	↓incidence; symptom improvement (65, 68)
Manual Lymphatic Drainage	Post-operative	Symptom Modulation	Symptom relief (69–71)
Complex Decongestive Therapy (CDT)	Post-diagnosis/early disease	Disease Stabilization	Symptom relief (69–71)
Compression-based Prophylaxis	Early post-operative	Primary Prophylaxis	↓ swelling; delayed disease onset (72, 73)
Immediate Lymphatic Reconstruction (LYMPHA/ILR)	Intra-operative	Primary Prophylaxis	↓ early BCRL incidence; ↓ procedure-related morbidity (75, 76)
Prophylactic Lymphatic Bypass	Intra-operative	Primary Prophylaxis	↓ incidence (selected patients) (77, 78, 80)
Axillary Surgery Optimization (SLNB vs. ALND)	Surgical planning	Risk Avoidance	↓ BCRL risk (81, 82)
Breast Cancer Reduction	Pre- and post-operative	Risk Reduction	↓ incidence (83–85)

CDT, Complex Decongestive Therapy; LYMPHA, lymphatic microsurgical preventive healing approach; ILR, Immediate lymphatic reconstruction; SLNB, sentinel lymph node biopsy; ALND, axillary lymph node dissection; BCRL, breast cancer-related lymphedema.

## Conservative management strategies

### Intermittent pneumatic compression

Intermittent pneumatic compression (IPC) is a method of conservative management used for BCRL, which includes applying sequential external pressure to the limb affected to enhance venous and lymphatic return (68). Previous clinical studies have shown that IPC can reduce limb volume and improve BCRL symptoms when combined with other standard treatment regimens, such as compression garments and exercise routine, and that IPC may also provide short-term limb volume reduction and symptom relief when compared to compression garments alone (85, 86). Recent reviews of IPC support its use with other management approaches but emphasize that evidence indicating its independent use is limited (77).

### Combined and multimodal management

Randomized and controlled trials support that combining CDT with additional physical rehabilitation regimens demonstrates beneficial effects regarding pain, limb-volume, and overall function when compared to single-modality treatments (87). Multimodal protocols incorporating compression, exercise, and MLD have shown improvements in upper-limb volume and function, though differences in protocols and outcomes measurement standards limit direct comparisons (88). Recent studies support that additional protocols incorporating low-level laser therapy and electrotherapy may provide additional patient benefits (89).

### Psychosocial interventions

Psychosocial interventions, such as education, coping strategies, mind-body practices, and personal well-being maintenance may improve overall QoL when integrated with physical lymphedema management strategies such as the ones discussed above (90). A qualitative study investigates the lived experience of women with BCRL, emphasizing the need for enhanced coping strategies, accessible mental health support, and the implementation of psychosocial interventions into management protocol (91). Prospective studies of postmastectomy care programs in Mexico consisting of structured patient education, physical rehabilitation, compression and physical care guidance, psychosocial support, and regular follow-up visits showed improvements in overall patient QoL, emotional and social functioning, and physical symptom burden relief (92).

## Surgical management approaches

### Overview of surgical hierarchy

Surgical management of BCRL is considered when conservative therapies are insufficient and is guided by disease severity, tissue characteristics, and individual patient factors (93). Surgical approaches are broadly categorized into physiologic procedures, which aim to restore or improve lymphatic drainage, and excisional procedures, which focus on removing pathologic tissue in more advanced disease (12). Physiologic techniques, such as lymphovenous anastomosis and vascularized lymph node transfer, are generally favored in earlier stages when functional lymphatic channels remain, whereas excisional procedures, including liposuction and radical reduction techniques, are typically reserved for more advanced or fibrotic disease (12). Increasingly combined surgical approaches and emerging techniques are being explored to optimize outcomes and tailor treatment to individual disease patterns.

## Physiological procedures

### Lymphaticovenous anastomosis

Lymphaticovenous anastomosis (LVA) is a microsurgical technique that restores lymphatic drainage by connecting functional lymphatic vessels to adjacent venules, allowing lymph to bypass obstructed pathways and re-enter the venous circulation (94). Successful outcomes depend on accurately identifying functional lymphatic channels and creating precise surgical connections that prevent venous backflow (94).

Advances in imaging have improved preoperative planning and intraoperative precision for LVA. Indocyanine green (ICG) lymphography is widely used to visualize functional lymphatic vessels and guide surgical planning (95). High-frequency and ultra-high-frequency ultrasound allow detailed visualization of lymphatic vessels and adjacent venules, facilitating accurate identification of suitable anastomosis sites and improving surgical planning (95). These imaging modalities may be used in combination to enhance lymphatic vessel mapping and optimize surgical outcomes (95). Contrast-enhanced ultrasound (CEUS) enables real-time visualization of lymphatic flow and vessel structure, allowing more accurate identification of lymphatic channels and recipient veins and improving overall surgical efficiency (94).

Clinical studies support the effectiveness of LVA in reducing limb swelling, decreasing episodes of cellulitis, and improving QoL in appropriately selected patients (94). As imaging and microsurgical techniques continue to advance, the LVA has become an increasingly refined and reproducible option within physiological surgical management of BCRL (94).

### Vascularized lymph node transfer

Vascularized lymph node transfer (VLNT) is a physiologic surgical option used for patients with BCRL, particularly those with ISL stage II and III disease or those who do not respond adequately to LVA, although studies have included patients across multiple ISL stages (94, 96). The procedure involves transferring functional lymph nodes from a donor site to the affected area to help restore lymphatic drainage and improve lymphatic function (94). A recent systematic review reported that VLNT has been performed in patients with ISL stage I (11.2%), stage II (71.3%), and stage III (17.5%) lymphedema (96).

Early clinical studies demonstrated that VLNT can reduce limb volume and improve lymphatic drainage in the affected extremities (94). Ongoing refinements in surgical technique and recipient-site selection have further improved outcomes, supporting its role as an effective option across multiple stages of lymphedema (94, 96). Clinical evidence suggests that VLNT may provide greater improvement in lymphedema severity compared with conservative therapy and LVA.

## Excisional procedures

### Liposuction

Liposuction is an excisional surgical approach used in patients with advanced or chronic BCRL, particularly when excess adipose and fibrotic tissue predominate and physiologic procedures are less effective (15). Because lymphedema involves progressive adipose deposition, liposuction aims to reduce limb volume by removing this pathologic tissue rather than restoring lymphatic flow (15).

Clinical studies have demonstrated that liposuction can achieve substantial and durable volume reduction, with sustained improvements observed years after treatment (15), with particular effectiveness for patients with non-pitting edema (96). Long-term follow-up studies by Brorson and colleagues have demonstrated near-complete and sustained limb volume reduction when liposuction is combined with controlled compression therapy, along with significant improvements in patient QoL and functional outcomes (25). Reductions in the frequency of soft-tissue infections and improvements in QoL have also been reported following the procedure (15, 97), because liposuction does not restore lymphatic function, long-term compression therapy remains essential to maintain outcomes (15).

Recent studies further support the effectiveness of liposuction for chronic lymphedema. In a multidisciplinary surgical model, patients undergoing power-assisted liposuction demonstrated significant and sustained reductions in limb volume, improved HRQoL, and decreased reliance on conservative therapies (97).

### Radical reduction

Radical reduction procedures are excisional surgical options typically reserved for patients with advanced BCRL who exhibit substantial fibrofatty tissue deposition that does not respond to physiologic techniques (98). These approaches aim to remove extensive pathologic tissue that contributes to persistent limb enlargement and impaired function.

Traditional methods, such as the Charles procedure and staged excisional techniques, historically reduced limb volume but were associated with complications including recurrent lymphedema, skin graft loss, and suboptimal cosmetic outcomes (98). Modern adaptations, such as radical reduction with perforator preservation, involve excision of deep fibrofatty tissue while maintaining blood supply to overlying skin flaps, offering durable volume reduction and improved aesthetic results (98). Although these procedures do not restore lymphatic function, they remain an important option for patients with advanced disease and irreversible tissue changes (98).

## Combined surgical approaches

Combined surgical approaches integrate both physiologic and reductive techniques to address the multifactorial nature of advanced BCRL (98). While physiologic procedures such as LVA and VLNT aim to restore lymphatic drainage, excisional techniques target the removal of accumulated fibrofatty tissue (98). In patients with advanced disease, combining these approaches allows for more comprehensive management than either strategy alone (98).

In addition to improving anatomic and functional outcomes, combined surgical approaches reflect a shift toward more individualized treatment planning in BCRL (98). Rather than relying on a single modality, treatment strategies can be tailored based on disease stage, tissue composition, and patient-specific goals (98). This personalized approach allows surgeons to address both lymphatic dysfunction and structural tissue changes within the same treatment framework, improving overall therapeutic flexibility (98). As surgical techniques and imaging modalities continue to evolve, combined approaches may further enhance outcomes by allowing more precise intervention while minimizing procedural burden (98).

## Innovative surgical techniques

### Omental flap–based reconstruction

Omental flap–based reconstruction (O-FAFF) is an emerging option for patients with advanced BCRL when physiologic procedures alone are insufficient (99). The omentum contains a rich lymphatic and vascular network that can support lymphatic regeneration and improve drainage when transferred to affected areas (99). When combined with fat augmentation, this approach may enhance both functional recovery and soft tissue restoration (99). Early clinical studies suggest that this approach can reduce limb volume and symptom burden while maintaining a low risk of complications at the donor site (99). Although long-term data remain limited, O-FAFF represents a promising adjunct within advanced and combined surgical management strategies for lymphedema (99).

### 3D modeling and robotic-assisted surgery

Robotic-assisted microsurgery is an emerging technology in the surgical management of BCRL that aims to improve precision, stability, and consistency in delicate lymphatic procedures (99). Highly specialized microsurgical robotic systems help overcome human limitations such as physiological tremor and fatigue, which can impact outcomes during super microsurgical lymphatic anastomoses (99). Clinical experience over the first several years of robotic use has included hundreds of robotic-assisted lymphatic anastomoses, with a substantial portion performed in patients treated for BCRL, demonstrating feasibility and expanding the technical reach of lymphatic reconstruction (99). While optimal indications and standardized protocols are still being defined, early data highlights the potential for robotic platforms to enhance operative precision and support complex lymphatic surgery in this population (99).

A summary of all conservative vs. surgical management options of BCRL is highlighted in **Table 2**.

**Table 2:** Conservative vs. surgical management of breast cancer-related lymphedema (BCRL).

Feature	Conservative management	Surgical management
Primary Role	First-line therapy for BCRL, particularly early or mild disease (69, 78, 88)	Utilized for refractory and/or advanced disease (94)
Main Goal	Symptom control, functional maintenance, and HRQoL improvement (78, 88, 91–93)	Structural correction and/or restoration of lymphatic drainage (12, 94)
Core Approaches	Compression garments, CDT, exercise, MLD, IPC, psychosocial interventions (69, 78, 88–93)	Physiologic procedures (LVA, VLNT) and excisional procedures (liposuction, radical reduction) (12, 99, 100)
Mechanism of Action	Enhances lymphatic and venous return; manages symptoms (69, 78)	Restores lymphatic flow (physiologic) or removes fibrofatty tissue in advanced disease (15, 99, 100)
Effectiveness by Disease Stage	Most effective in early-stage or less fibrotic disease (9, 11, 78)	Stage-dependent: physiologic procedures favored earlier; excisional procedures used for advanced and/or fibrotic disease (12, 94, 99)
Impact on Limb Volume	Modest and often temporary reductions (78, 86, 87)	Substantial and durable volume reduction, especially with excisional procedures (15, 98, 99)
Effect on Lymphatic Function	Does not restore lymphatic anatomy or function (78)	Physiologic procedures aim to restore or improve lymphatic drainage; excisional procedures do not (99, 100)
HRQoL Benefits	Improves pain, function, emotional well-being, and coping when integrated with physical therapy (88, 91–93)	Improves limb size, infection burden, and long-term functional outcomes (97, 98, 100, 101)
Durability of Results	Requires ongoing adherence to therapy and compression (78, 88)	Long-lasting volume reduction; lifelong compression often required after excisional surgery (15)
Limitations	Limited effectiveness in advanced disease; high patient burden and need for sustained adherence (78, 89)	Requires specialized expertise, operative resources, and individualized planning (94, 99, 101)

HRQOL, health-related quality of life; BCRL, breast cancer-related lymphedema; CDT, Complex Decongestive Therapy; MLD, Manual lymphatic drainage; IPC, Intermittent pneumatic compression; LVA, Lymphaticovenous anastomosis; VLNT, Vascularized lymph node transfer.

## Emerging and experimental approaches

### Stem cell-based regenerative therapies for BCRL

**Figure 4:**
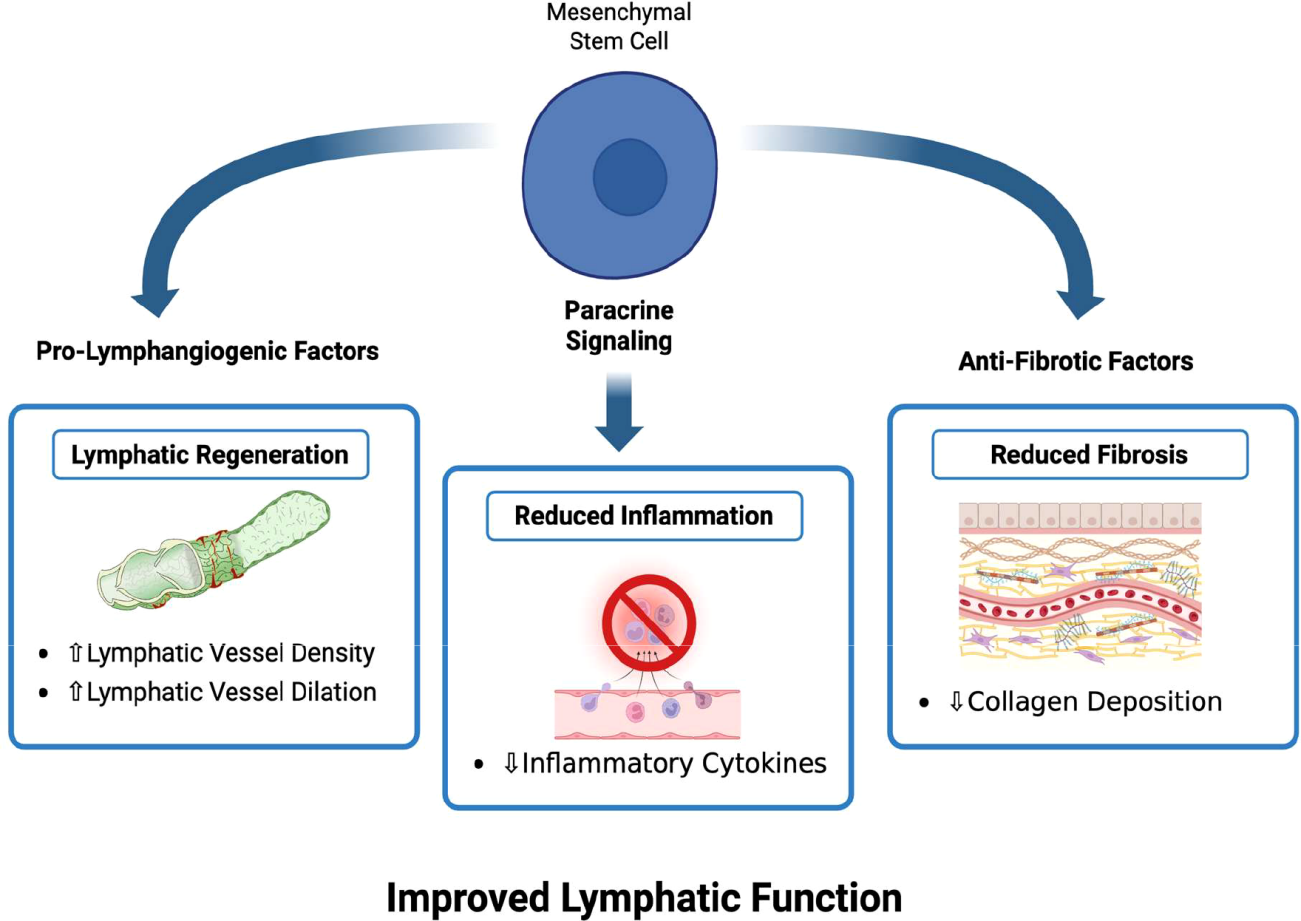
Schematic representation of mesenchymal stem cell-based regenerative approach in breast cancer-related lymphedema (BCRL).

While current therapeutic strategies for BCRL, such as physiotherapy and surgery, can alleviate symptoms, they often do not restore normal lymphatic function or fully halt disease progression, particularly in advanced disease (101, 102). As a result, regenerative approaches such as stem cell-based therapies have gained interest as experimental strategies because of their proposed potential to support lymphatic repair and modulate the inflammatory and fibrotic milieu underlying lymphedema (101). Pre-clinical and early translational studies suggest that mesenchymal stem cells (MSCs), including adipose- and bone marrow-derived MSCs, may exert their effects in part through paracrine secretion of pro-lymphangiogenic growth factors (102). **Figure 4** illustrates the proposed mechanisms underlying MSC regenerative approaches in BCRL, including paracrine signaling that may promote lymphangiogenesis, reduce inflammation, and limit fibrosis (**Figure 4**). These pathways are thought to contribute to improved lymphatic function; however, these strategies remain investigational and require further validation in clinical studies.

In a small early-phase clinical study, autologous bone marrow stromal cell (BMSC) transplantation in women with BCRL was associated with greater long-term reductions in pain and limb edema compared with CDT, despite similar early outcomes (103). In that study, BMSC treatment was associated with greater pain reduction at 12 months (*p* = 0.0353) and reductions in limb edema volume through 12 months (*p* = 0.0001) (103). In a preclinical rabbit model of secondary limb lymphedema, combining BMSC transplantation with vascular endothelial growth factor-C (VEGF-C) was associated with greater improvement than BMSC or VEGF-C monotherapies, suggesting a possible synergistic effect on lymphatic regeneration (104). Adipose-derived stem cells (ADSCs) have also shown promise in experimental models of lymphedema. In a secondary lymphedema mouse model, ADSC transplantation was associated with increased lymphatic vessel density, improved lymphatic vessel dilation, and reduced tissue fibrosis (105). In preclinical murine models of secondary lymphedema, localized administration of ADSC-derived extracellular vesicles (EVs) was associated with reduced limb swelling and increased capillary and lymphatic vessel density, supporting a proposed paracrine, cell-free contribution to lymphatic repair (102). Together, these findings suggest that stem cell-based regenerative strategies may promote lymphatic regeneration in preclinical models. However, the evidence remains largely limited to animal studies and early-phase clinical data. These approaches therefore remain experimental and require validation in large-scale, controlled trials prior to clinical application.

### Tissue engineering approaches to lymphatic reconstruction

Tissue engineering approaches represent an emerging regenerative strategy aimed at supporting lymphatic regeneration through structural support. Biomaterial scaffolds are being explored as experimental tools to promote organized lymphangiogenesis, particularly in fibrotic or surgically scarred tissue (106). Aligned nanofibrillar collagen scaffolds, such as BioBridge™, have been shown in preclinical models to support directional lymphatic regeneration by providing structural guidance across disrupted lymphatic channels (106). In a preclinical rat lymphedema model, scaffold implantation was associated with reduced lymphedema development following lymphadenectomy and improvements in established disease (106). In a preclinical porcine model of secondary lymphedema, scaffold implantation was associated with increased lymphatic vessel ingrowth across scarred tissue when compared with control interventions (107). Notably, scaffold-mediated regeneration occurred both alone and in combination with autologous lymph node fragments, suggesting that structural guidance may facilitate directed lymphangiogenesis even in fibrotic environments (107). Although most evidence for scaffold-based lymphatic regeneration is derived from preclinical models, clinical data remain sparse. Early human studies, including small retrospective analyses and case reports suggest potential benefit when these approaches are incorporated into established surgical treatments such as ALNT, LVA, or liposuction in selected patients (108). However, these findings remain preliminary and require validation in larger, controlled studies before broader clinical application.

### Drug-based experimental therapies targeting inflammation and fibrosis

Given the central role of inflammation and fibrosis in lymphedema pathophysiology, pharmacologic strategies targeting these pathways are being explored as adjunctive approaches, primarily in preclinical and early translational settings (109). In preclinical mouse models of lymphedema, pharmacologic inhibition of the profibrotic cytokine transforming growth factor-beta 1 (TGF-β1) has been associated with improvement in fibrotic and inflammatory tissue changes. For example, topical administration of pirfenidone, an anti-fibrotic agent that suppresses TGF-β1 signaling, reduced tissue fibrosis, and decreased lymphedema formation in animal models (110). Limited clinical evidence has also emerged for anti-inflammatory pharmacologic approaches in lymphedema. In a placebo-controlled trial, treatment with the nonsteroidal anti-inflammatory drug ketoprofen reduced skin thickness and improved histopathologic measures of inflammation (111). However, ketoprofen did not produce a significant increase in micro-lymphatic vascular area when compared with control, suggesting that reduction of inflammation may not necessarily translate into measurable lymphatic regeneration (111). Overall, pharmacologic modulation may improve certain pathologic features of lymphedema; however, current evidence is limited and largely preclinical, and requires validation in larger clinical studies.

### Immunomodulatory therapies promoting lymphatic regeneration

Tacrolimus is a calcineurin inhibitor that targets T-cell-mediated inflammation and has been investigated for potential effects on lymphatic regeneration. By inhibiting T-lymphocyte activation and proliferation, tacrolimus targets upstream pathways involved in tissue fibrosis and impaired lymphangiogenesis (112). In preclinical mouse models, topical tacrolimus reduced swelling, T-cell infiltration, and tissue fibrosis, and was associated with increased lymphatic collateral vessel formation and improved collecting vessel function (112). These findings suggest a combined anti-inflammatory and lymphatic-supportive effect, in contrast to agents that primarily target inflammation or fibrosis. In a recent multicenter pilot study of breast cancer patients undergoing axillary lymph node dissection, topical tacrolimus 0.1% ointment did not significantly reduce lymphedema incidence at 12 months. However, exploratory analyses suggested possible improvements in symptom burden and quality of life, which highlights the need for further evaluation in larger randomized control trials (113).

## Limitations

The current narrative review is not without limitations. As with any narrative review, there is inherent bias in the selection of articles included in the manuscript (114). In order to mitigate this bias, the authors queried the PubMed, Embase, and SCOPUS databases using a Boolean search string including keywords such as “breast cancer,” “lymphedema,” “management,” and “treatment.” Included articles were published from database inception to October 2025, with a particular emphasis placed on selection of articles from the last five years (2020–2025). Articles were included if they discussed management, incidence, or otherwise furthered the reader’s current understanding of breast cancer-related lymphedema. Exclusion criteria was at the authors’ discretion as to whether the article addressed the main purpose of the present review. Particular emphasis was placed on inclusion of higher level of evidence articles (Randomized controlled trials (RCTs), prospective comparative studies, and meta-analyses). Future studies should strive to adopt a systematic format in addressing outcomes of particular management modalities for breast cancer-related lymphedema; however, the present study serves as a comprehensive and highly-descriptive review of our current understanding of these modalities.

## Conclusion and future perspectives

The persistent healthcare burden presented by BCRL represents a source of significant concern. The current standards of care in the diagnosis and treatment of breast cancer continue to pose a risk of post-treatment lymphatic disruption. This review highlights the current peer-reviewed literature reporting on the mechanisms, clinical features, diagnostics, risk assessment and interventions related to BCRL utilizing multidisciplinary approach.

Risk factors leading to BCRL are abundant, with ALND presenting as the highest risk. Other factors include lymph node dissection positivity, high BMI, use of taxane-based chemotherapy, and regional lymph node radiation. BCRL presents early with swelling, heaviness, and pressure, often with a visibly appreciative size discrepancy of the affected limb. The painful swelling associated with BCRL not only presents a clinical disturbance to the patient, but also a psychological one, as there are both cosmetic and functional considerations affecting the patient’s HRQoL. Diagnostic and staging of BCRL is multimodal and may include clinical examination, subjective reporting of symptoms, circumferential measurement, and imaging modalities such as ultrasound; however, specific tools used may vary based on institutional protocols and available resources.

While there are numerous surgical modifications and preventative techniques detailed in this article to prevent BCRL, the summation of the current peer-reviewed literature suggests that a multimodal approach to prevention and treatment is the current best practice. Integration of the techniques listed in this article provides the best opportunity for the reduction of the clinical burden presented by BCRL. As we continue to optimize these techniques using available and future data, we can move towards a complete reduction of symptoms and preservation with improved HRQoL for those affected by BCRL.
